# Uncover the Offensive Side of Disparagement Humor: An fMRI Study

**DOI:** 10.3389/fpsyg.2021.750597

**Published:** 2021-11-22

**Authors:** Angela Bartolo, Daniela Ballotta, Luca Nocetti, Patrizia Baraldi, Paolo Frigio Nichelli, Francesca Benuzzi

**Affiliations:** ^1^Univ. Lille, CNRS, UMR 9193 - SCALab - Sciences Cognitives et Sciences Affectives, Lille, France; ^2^Institut Universitaire de France (IUF), Paris, France; ^3^Dipartimento di Scienze Biomediche, Metaboliche e Neuroscienze, Università di Modena e Reggio Emilia, Modena, Italy; ^4^Fisica Medica, Azienda Ospedaliera Universitaria di Modena, Modena, Italy

**Keywords:** disparagement humor, social inappropriateness, offense, event-related design, emotions, humor, fMRI

## Abstract

Disparagement humor is a kind of humor that denigrates, belittles an individual or a social group. In the aim to unveil the offensive side of these kinds of jokes, we have run an event-related fMRI study asking 30 healthy volunteers to judge the level of fun of a series of verbal stimuli that ended with a sentence that was socially inappropriate but funny (disparagement joke -DJ), socially inappropriate but not funny (SI) or neutral (N). Behavioral results showed disparagement jokes are perceived as funny and at the same time offensive. However, the level of offense in DJ is lower than that registered in SI stimuli. Functional data showed that DJ activated the insula, the SMA, the precuneus, the ACC, the dorsal striatum (the caudate nucleus), and the thalamus. These activations suggest that in DJ a feeling of mirth (and/or a desire to laugh) derived from the joke (e.g., SMA and precuneus) and the perception of the jokes’ social inappropriateness (e.g., ACC and insula) coexist. Furthermore, DJ and SI share a common network related to mentalizing and to the processing of negative feelings, namely the medial prefrontal cortex, the putamen and the right thalamus.

## Introduction

In recent years, there has been increasing interest in the neural substrate of humor, in both basic neuroscience and clinical studies. Many authors have focused on aspects of humor comprehension and appreciation, using semantic and phonological jokes (Coulson and Kutas, 2001; Goel and Dolan, 2001; Coulson and Severens, 2007), cartoons (Gardner et al., 1975; Wapner et al., 1981; Shammi and Stuss, 1999; Mobbs et al., 2003, 2005; Bartolo et al., 2006; Wild et al., 2006; Goel and Dolan, 2007; Samson et al., 2008), or movies (Iwase et al., 2002; Moran et al., 2004; for a review see Wild et al., 2003).

In this study, we focused on disparagement humor, a kind of humor that elicits amusement through denigration, as it defames, belittles, or maligns an individual or a social group (Janes and Olson, 2000; Ferguson and Ford, 2008; McGraw and Warren, 2010; Koszałkowska and Wróbel, 2019). Psychoanalytic and superiority theories, as they focus on context, are supposed better providing an explanation to disparagement humor (Ferguson and Ford, 2008). However, while the former considers disparagement humor as a benign means of expressing socially unacceptable impulses, the superiority theory sustained that humiliation is the central component of humor: in the process of getting fun from ridiculousness, our own sense of superiority receives an unexpected boost, humor acts as a sort of instrument that inflates our own ego and deflates that of others, “humor entangled with hatred encourages a sense of moral superiority” (Sakki and Martikainen, 2021, p. 610). In this vein, to laugh at someone it is necessary not to be emotionally involved with the person subjected to offense. It is necessary to shut pietas and sympathies and to look at the situation as external spectators: “to produce its effect, the comic needs a temporary anesthesia of the heart” (Bergson, 1900, p. 6). It follows that the inability to anesthetize hearth could make the joke not funny at all.

Since the core of disparagement humor is the insult, its disapproval in the public domain seems to be based on the belief that such humor might have negative consequences. Specifically, it is thought to create and reinforce stereotypes of social groups and perpetuate prejudice (Ford and Ferguson, 2004). It is not surprising, therefore, that this type of humor is of interest to social psychology and criminology (Ford and Ferguson, 2004).

In disparagement humor, an individual or a category of people is typically portrayed in a ridiculous manner. Consider this joke deriding the category of engineers: *“An engineer is on his first day at work. When he arrives, his boss gives him a broom and asks him to clean the floor. The guy protests “But I am an engineer!” “Ah, you’re right,” the boss replies, “I’ll show you how it works.”* In this joke, the category of engineers is targeted, and the perceiver must cope with two different and contrasting processes: the feeling of mirth derived from the comprehension of humor and the perception of offense. According to the *Incongruity-Resolution Model* (Suls, 1972) humor comprehension is critically dependent upon resolving incongruity between the punch line and expectations shaped by the storyline. According to this theory, there are two cognitive stages for humor comprehension: detection and resolution of incongruity. Incongruity is generated when the prediction that rose in the first part of a story is not confirmed in the final part, generating an incongruous statement. To comprehend humor, it is necessary to revisit the story and reconcile the incongruity by transforming the incongruous statement into a funny congruent story. In the example above, the engineer is asked to clean the floor. By saying “*But I am an engineer*!” one understands that this activity is perceived by the engineer as inappropriate to his social status. However, in the punch line, the boss says something unexpected and apparently incongruous: “*Ah, you’re right*; *I’ll show you how it works.*” “*Ah, you’re right*” does not mean, as expected, that the boss has understood that the work is inappropriate to the engineer’s social status. To understand the punch line (i.e., “*I’ll show you how it works*”), one needs to reconsider the meaning of “*Ah, you’re right*” and attribute to it another meaning to make sense of the joke: “the boss thinks that the engineers are good-for-nothing.” Once the incongruity is solved, the sentence makes sense (resolution of incongruity) and a feeling of amusement might follow. However, to laugh at a joke that belittles someone else might be socially inappropriate. By getting the joke, one understands that the person is belittled, therefore, if one admits that the joke is funny, it is like accepting that someone is insulted. In this sense, disparagement humor might generate a feeling of embarrassment and guilt (Ferguson and Ford, 2008).

In the case of the engineer, one can assume that his consideration toward the job of cleaning the floor was somehow arrogant. Considering his educational level, the person evaluated that this type of job should be done by people with low education. This can be judged as haughty and arrogant; therefore, the character shows negative qualities. This joke acts as a way to lessen the arrogance of the protagonist in a playful way. In other words, “castigat ridendo mores” (“laughter corrects customs/manner,” Jean de Santeul 1630–1697), meaning that by pointing out the absurdity of customs and laughing at them, things can change.

Chan et al. (2016) studied hostile jokes, defined as “sarcastic expressions of aggression,” using fMRI. This kind of joke is like disparagement humor, with the difference that the former targets a particular social group. They found activations in the midbrain, the left dorso-medial prefrontal cortex (dmPFC), the left ventro-lateral prefrontal cortex (vlPFC), and the left insula in hostile jokes relative to either non-hostile jokes or neutral sentences. The increased activation in the left dmPFC and left vlPFC was interpreted as related to the comprehension of the hostile intentions, whereas the activation in the midbrain and left insula was ascribed to the appreciation of the hostile aggression (Chan et al., 2016). However, Chan et al. (2016) did not show in their study the brain activity during the processing of hostile sentences and did not explore if hostile jokes and hostile sentences shared common activations.

The present study aimed to investigate the brain regions engaged specifically for disparagement humor and to evaluate commonalities and differences between the brain network subserving disparagement humor and socially inappropriate but not funny sentences. To this end, in an event-related fMRI study, we asked 30 healthy volunteers to judge the level of fun of 40 verbal stimuli that were socially inappropriate but funny (i.e., disparagement joke -DJ), socially inappropriate but not funny (i.e., offensive, SI) or neutral (N).

We make two hypotheses: one holds that to perceive disparagement humor, participants have to ignore the offensive parts of the joke and focused on the funny parts; therefore, only SI should activate the brain areas associated with the perception of the offense. Reversely, if participants are amused but at the same time they perceive the offense in DJ, then, both DJ and SI should activate the brain areas associated with the perception of the offense.

## Materials and Methods

### Subjects

Thirty right-handed healthy volunteers (15 females, Range_age_ = 20–44 years; *M*_*age*_ = 28.9 yrs, *SD*_*age*_ = 6.0 yrs; *M*_*education*_ = 14.7 yrs, *SD_*education*_* = 2 yrs) participated in the fMRI study. Handedness was determined by means of the Edinburgh Inventory Scale (Oldfield, 1971). All the subjects had normal or corrected-to-normal visual acuity and gave formal consent to participate in this study. The subjects were paid for their participation or received university credits. This study was approved by the local Ethics Committee.

### Stimuli and Experimental Design

Thirty-two disparagement jokes (DJ) were chosen from different internet websites. They contained different types of jokes concerning gender, human race, authorities, politicians, religions.

Jokes were modified in a way that each of them consisted of two sentences: a set up line and a funny punch line. Using the same set up lines, an equal number of SI and N stimuli were generated by replacing the punch line:

Example:
SET UP LINE:
*A prostitute to another one: “What did you ask Santa Claus?*
PUNCH LINE:
  (1) Funny response (DJ): 5*0€, like everyone else!*
  (2) Socially inappropriate response (SI): *Santa Claus is a lousy old man!*
  (3) Neutral response (N): *A better life!*

To select the set of stimuli to be presented during the fMRI sessions, a group of 15 volunteers, different from those subjected to the fMRI experiment, were asked to rate the 96 stimuli (32 stimuli X 3 types: DJ, SI, and N) on a scale of 0 (very offensive) to 6 (very funny). A rating of 3 indicated that the stimulus was neutral. Twenty stimuli reached on average a score of 4.5 or higher and were included in the set of disparagement jokes. Twelve stimuli were rated on average 1.5 or lower and were included in the set of offensive items. Eight stimuli reached a mean score of 3 and were included to form the set of neutral stimuli.

**FIGURE 1:**
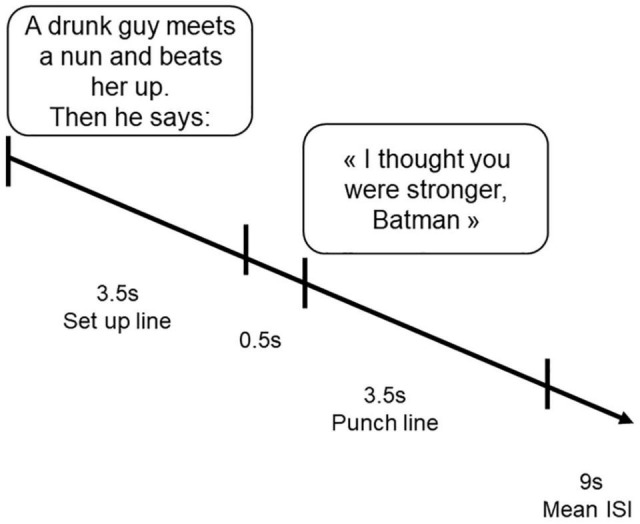
A typical trial making up each session. Stimuli could be disparagement jokes, offensive and neutral sentences. The setup line begun at time 0 and remained on the screen for a duration of 3.5 s. A 0.5 s-interval elapsed between the presentation of the two sentences making up each pair. The punch line remained on the screen for other 3.5 s. The inter stimulus interval (ISI) was varying between 2.5 and 20.5 (Mean ISI = 9 s).

The fMRI experiment was carried out using an event related design (see Figure 1). Each subject performed two fMRI runs, each including the presentation of 10 DJ, 6 SI, and 4 N for a total of 40 stimuli. Each stimulus comprised a setup line lasting 3.5s, followed after 0.5s by the punch line lasting 3.5s (DJ, SI, or N). Subjects were instructed to indicate the level of fun of each stimulus along a four-point scale (0 = not funny; 3 = very funny) at the presentation of the punch line stimulus (see Figure 1), by pressing one of four response keys, using the thumb, the index, the medium, and the ring finger, respectively. For half of the subjects the scale was ascending from thumb (very funny) to the ring finger (not funny at all), the other half the scale was reversed. Stimuli were counterbalanced across runs, and the presentation was randomized. Each run lasted for 5 min.

Stimuli were presented using the IFIS-SA system (MRI Devices Corp., Waukesha, WI). E-prime (Psychology Software Tools, Inc.)^1^ was used to control stimulus delivery and to record behavioral responses (type of answer and reaction times). To acquaint subjects with the task, they rehearsed a couple of items before the experimental phase.

Following the acquisition session, out of the scanner, the subjects were asked to rate each of the 40 stimuli on the level of offense they experienced, using a scale ranging from 0 (not offensive) to 3 (very offensive). They were asked to base their answers on how they had perceived the stimuli while they were inside the scanner.

### fMRI Data Acquisition

MRI data were obtained on a 3 T Philips Gyroscan Intera MR Scanner (Philips Medical Systems), using the standard setup of body coil transmission and SENSE head coil reception.

BOLD-sensitive fMRI images were acquired using a T2*-weighted gradient-echo echo-planar sequence (TR = 2,000 ms, TE = 35 ms, FA = 80°, FOV = 240 mm, matrix 64 × 64, SENSE factor = 2) to obtain thirty axial slices of 4 mm thickness with no interslice gap, covering the whole brain. A total of 150 volumes were collected in each scan. A high-resolution T1-weighted 3D gradient-echo anatomical image was also acquired to allow anatomical localization (TR = 9,900 ms; TE 4.6 ms; FOV: 256 MM; matrix: 256 × 256; voxel dimension 1.0 × 1.0 × 1.0). Subjects were instructed to refrain from laughter to reduce movement artifacts. Head motion was minimized through foam padding within the head coil.

### Data Analysis

Behavioral results have been analyzed by comparing the mean scores/response times among the three types of stimuli (DJ, SI, and N).

fMRI data analyses were performed using MatLab version R2020a (Mathworks, Natick, MA, United States) and SPM12 software (The Wellcome Centre for Human Neuroimaging, UCL Queen Square Institute of Neurology, London, United Kingdom). All functional volumes for each subject were slice-time corrected, realigned to the first volume acquired, and normalized to the MNI (Montreal Neurologic Institute) template implemented in SPM12. Then the images were smoothed with a Gaussian kernel having FWHM of 8 × 8 × 8 mm.

Single-subject statistical analysis was performed applying the general linear model, where the time-series data were modeled as a series of events convolved with a canonical hemodynamic response function. Three types of events were implemented as regressors in the single subject-first level analysis: DJ, SI, and N. For this event-related analysis, the appearance of the punch line was considered as the starting time of the stimulus of interest. The six head-motion parameters (translations and rotations) were entered as regressors of no interest.

Using statistical maps generated by the single-subject analyses, several random effect second level group analyses (One-Sample *t*-test) were performed as follows:

(a) Activations associated with DJ; contrasts DJ vs. baseline, DJ vs. N and DJ vs. SI;
(b) Activations associated with SI; contrasts SI vs. baseline, SI vs. N and SI vs. DJ.

To evaluate cortical activations common to DJ and SI three different regions of interest (ROIs) were defined using the SI > N contrast: right thalamus (peak coordinates: x = 2, y = 4, z = 6), left putamen (peak coordinates: x−30, y = −4, z = 2) and left medial frontal gyrus (peak coordinates: x−2, y = 52, z = 10; uncorrected for multiple comparisons, k > 0). From these ROIs we have extracted the beta values using Marsbar^2^ for the DJ and N conditions and we have run a *t*-test over them.

A double statistical threshold (voxel-wise *p* < 0.001 and spatial extent) was adopted to achieve a combined significance, corrected for multiple comparisons, of α < 0.05, as computed by 3dClustSim AFNI routine, using the ‘‘-acf’’ option^3^.

## Results

### Behavioral Results

**FIGURE 2:**
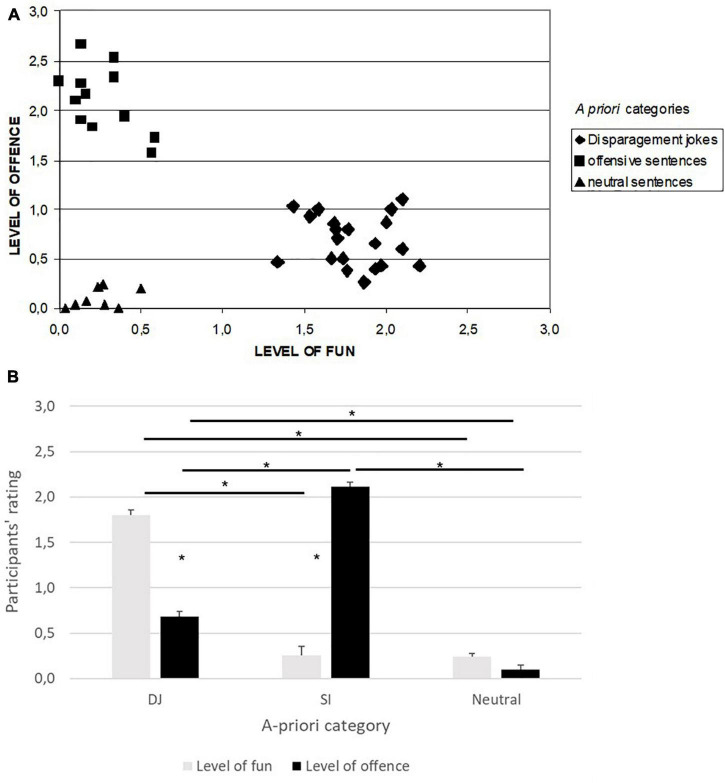
Stimulus rating given by the thirty participants along two dimensions: fun and offensive. **(A)** Each point represents the average of the answers given by the subjects for each of the forty stimuli for the two dimensions. Different symbols indicate the three *a priori* classes of the presented stimuli: Neutral (triangle) and Offensive (square) sentences, and Disparagement Jokes (rhombus). **(B)** Participants’ rating on the level of fun and offense in the three a-priori categories. *indicate significant differences.

The average scores given by the participants to each stimulus according to the three a-priori categories (DJ, SI, and Neutral) are reported in Figure 2A for the two rated dimensions (level of fun, level of offense). As shown in Figure 2A, on average subjects rated the stimuli according to the a-priori categories.

By considering the three a-priori categories of stimuli and by using data collected inside (level of fun) and outside of the scanner (level of offense), results on skewness and kurtosis revealed that data distribution was normal in each category of stimuli (for response time and for the two levels of rating - fun and offense).

The one-way ANOVA on response time registered in the three a-priori categories did not reach a significant difference [*F*_(2, 37)_ = 2.034, *p* = 0.145]. Overall, participants took the same time to respond to DJ, SI and Neutral stimuli.

We run a 3 × 2 ANOVA for repeated measures on the 3 a-priori categories and according to the two levels of rating (fun and offense, see Figure 2B). Results showed a significant effect of the category [*F*_(2, 37)_ = 173.87, *p* < 0.001, η*_*p*_*^2^ = 0.90]. Tukey’s *post hoc* test showed that the category of Neutral stimuli differed from both DJ and SI (*p* < 0.001 in both cases). The difference between DJ and SI was not significant. The ANOVA also showed a significant effect of the levels of rating [*F*_(1, 37)_ = 9.127, *p* = 0.005, η*_*p*_*^2^ = 0.20], the rating was higher for the level of offence than for the level of fun; and a significant interaction type X rating [*F*_(2, 37)_ = 227.6, *p* < 0.001, η*_*p*_*^2^ = 0.92].

Tukey’s *post hoc* test showed a significant difference between level of fun and offense in DJ and SI (*p* < 0.001). In Neutral stimuli, the level of fun and offense did not differ (*p* = 1). Furthermore, in DJ the level of fun differed from that registered in SI stimuli (*p* < 0.001, it was higher in DJ), the same difference was found in the level of offense, which was higher in SI stimuli with respect to DJ (*p* < 0.001). The level of offense also differed in DJ with respect to Neutral stimuli (*p* < 0.001, it was higher in DJ). No difference in the level of fun between SI and Neutral was registered (*p* = 1). In summary, DJ stimuli were funny and at the same time offensive (the level of fun and offense in DJ differed from the two levels registered in Neutral stimuli); whereas SI stimuli were only offensive (no difference between the level of fun registered in SI and Neutral stimuli). DJ stimuli, although offensive, were rated as less offensive than SI stimuli (see Figure 2B).

### fMRI Results

#### Disparagement Jokes

DJ activated a large brain network including the bilateral cerebellum, the supramarginal gyrus (SMG), the hippocampus, the amygdala, the anterior and posterior insula, the inferior frontal (IFG) and postcentral gyrus (including the primary, S1, and secondary somatosensory cortex, S2), the thalamus and caudate nucleus, the anterior and mid-cingulate cortex, and the middle and inferior frontal gyri (Table 1).

**TABLE 1:** Significant areas of activation for the comparison “DJ vs. baseline” (cluster size threshold k ≥ 1, corrected at α < 0.05).

		Side	Cluster	Voxel level	MNI coordinates		
Brain areas	BA		K	T	x	y	z
Cerebellum, supramarginal gyrus, lingual gyrus, hippocampus, amygdala, anterior and posterior insula, inferior frontal gyrus, postcentral gyrus, thalamus, caudate nucleus	18, 40, 47	L/R	2,804	11.19	14	−56	−18
				9.65	62	−20	30
				9.55	2	−80	−14
Anterior and mid-cingulate cortex, middle and inferior frontal gyrus	24, 32	L/R	899	9.88	2	40	14
				7.48	42	52	10
				7.20	2	28	38
Thalamus, caudate nucleus		R	26	5.05	14	−4	14

*R, right; L, left; BA, Brodmann area.*

The comparison between DJ vs. N showed activation in the left anterior insula, the left entorhinal cortex and the left putamen (Table 2).

**TABLE 2:** Significant areas of activation for the comparison “DJ vs. N” (cluster size threshold k ≥ 24, corrected at α < 0.05).

	Side	Cluster	Voxel level	MNI Coordinates		
Brain areas		K	T	x	y	z
Anterior insula	L	31	3.50	−38	8	−2
Entorhinal cortex			3.51	−30	4	−22
Putamen			3.66	−30	0	−6

*R, right; L, left; BA, Brodmann area.*

**FIGURE 3:**
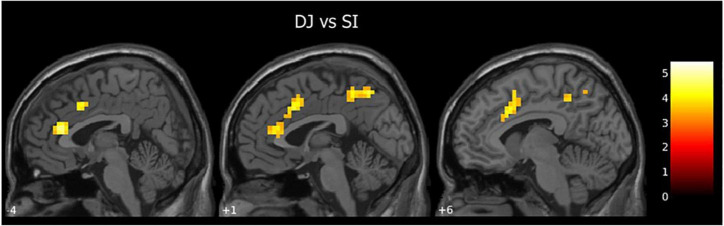
Second level group analyses. Brain areas activation associated with DJ (DJ vs. SI contrast; cluster size threshold k > 24, corrected at α < 0.05). Activation are superimposed on the SPM12 template.

When contrasting DJ vs. SI we found activations in the left anterior cingulate cortex (ACC, BA 24), and bilaterally in the supplementary motor area (SMA), the anterior and middle cingulate cortex (BA 24, 32) and the precuneus (BA 31; Table 3 and Figure 3).

**TABLE 3:** Significant areas of activation for the comparison “DJ vs. SI” (cluster size threshold k ≥ 24, corrected at α < 0.05).

		Side	Cluster	Voxel level	MNI Coordinates		
Brain areas	BA		K	T	x	y	z
Middle frontal gyrus	6	R	28	5.47	34	0	46
Anterior cingulate cortex	24	L	29	4.88	−2	32	18
Precuneus	31	R/L	39	4.47	2	−60	54
Supplementary motor	24, 32	R/L	51	4.42	−6	12	42
area, anterior and middle cingulate cortex				4.33	2	16	38

*R, right; L, left; BA, Brodmann area.*

#### Socially Inappropriate-Not Funny Stimuli

The main effect of SI showed activity in a large network including frontal (inferior, superior, medial frontal gyrus, ACC), parietal (SMG, and angular gyrus, AG) and temporal regions (superior temporal gyrus, STG), as well as the anterior and posterior insula, the bilateral amygdala, the hippocampus, the parahippocampal gyrus and the cerebellum (Table 4).

**TABLE 4:** Significant areas of activation for the comparison “SI vs. baseline” (cluster size threshold k ≥23, corrected at α < 0.05).

		Side	Cluster	Voxel level	MNI Coordinates		
Brain areas	BA		K	T	x	y	z
Inferior frontal gyrus	47	L	47	8.84	−46	44	−6
Cerebellum, lingual gyrus	18, 19	L/R	825	7.77	10	−60	−14
				7.47	6	−80	−14
				6.90	34	−76	−34
Supramarginal gyrus, inferior frontal gyrus, anterior and posterior insula, pre- and post-central gyrus	40, 44, 47	R	401	7.37	62	−24	26
				6.84	50	40	−10
				5.59	58	12	−2
Superior frontal gyrus, medial superior frontal gyrus, anterior cingulate cortex	8, 9, 32	L/R	404	6.84	−2	48	26
				5.52	−22	56	22
				5.48	−6	48	46
Post-central gyrus, supramarginal gyrus, angular gyrus, superior temporal gyrus, anterior and posterior insula, hippocampus, amygdala	22, 38, 40, 42	L	624	6.81	−58	−32	38
				6.46	−46	16	−10
				6.39	−62	−32	30
Parahippocampal gyrus, amygdala	28, 34	R	24	5.64	10	−8	−18
				4.07	26	4	−22
Middle frontal gyrus	9	R	45	5.38	42	20	46
				5.17	42	24	38

*R, right; L, left; BA, Brodmann area.*

SI vs. N evoked activations in the right caudate nucleus and thalamus (Table 5).

**TABLE 5:** Significant areas of activation for the comparison “SI vs. N” (cluster size threshold k ≥22, corrected at α < 0.05).

	Side	Cluster	Voxel level	MNI coordinates		
Brain areas		K	T	x	y	z
Caudate nucleus, thalamus	R	22	3.50	2	4	6

*R, right; L, left; BA, Brodmann area.*

The comparison of SI vs. DJ did not lead to significant activation at the selected statistical threshold.

#### Common Regions Between Disparagement Humor and Socially Inappropriate but Not Funny Jokes (Regions of Interest Analysis)

**FIGURE 4:**
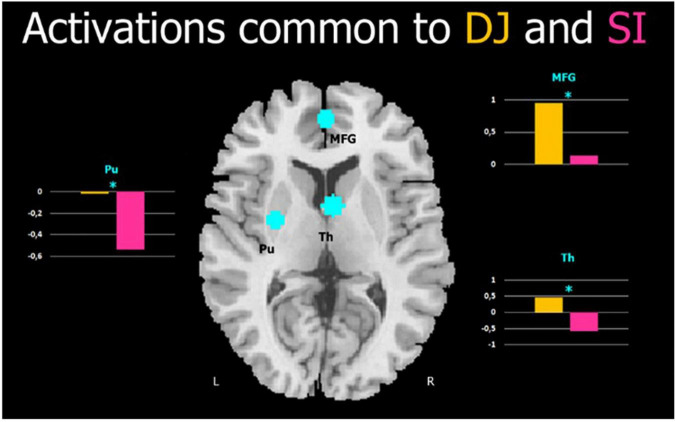
Cortical activation common to DJ and SI. Cyan blobs represent the three functional ROIs from the SI > N contrast (anatomical template found in xjview toolbox). Plots represent mean beta values for each ROIs and each condition (DJ and N); MFG, Medial Frontal Gyrus; Th, Thalamus; Pu, Putamen; L, left; R, right. *indicate significant differences.

A significant difference between DJ and N was found in the three ROIs: the right thalamus, *t*(29) = 3.8, *p* < 0.001, the left putamen, *t*(29) = 3.45, *p* < 0.002), and the left medial frontal gyrus, *t*(29) = 3.9, *p* < 0.001 (see Figure 4).

## Discussion

This study aimed at exploring the neural substrates of disparagement humor (DJ) in comparison to socially inappropriate but not funny jokes (SI) and to investigate to what extent DJ and SI are similar. During our event-related fMRI study, healthy volunteers were asked to judge the level of fun of a series of verbal stimuli that were socially inappropriate but funny (i.e., disparagement humor, DJ), socially inappropriate but not funny (SI) or neutral (N). Out of the scanner, the same participants were asked to rate the level of offense perceived for each stimulus while they were inside the scanner.

Behavioral results show that participants rated the stimuli as expected, ending up to three categories: DJ, SI, and N. In particular, SI were judged as funny as N stimuli, thus not funny, and they were judged as more offensive than DJ and N stimuli. Overall, SI stimuli well represent the category of offensive stimuli. DJ were funnier and more offensive than N stimuli, proving that the DJ stimuli well represent the category of disparagement jokes. However, DJ were perceived as less offensive than SI. This result might be explained by considering the nature of DJ that are at the same time funny and offensive: judging funny a joke that offends someone generates cognitive and moral conflict. In line with this view, it could be that individuals *de facto* perceived the offense, however, to preserve themselves from negative social-moral consequences (Janes and Olson, 2000) or to protect their ethical reputation (Higgins, 1987; Higgins and Bargh, 1987); they judged the jokes not very offensive. This result is in line with some comments that our subjects made out of the scanner, when they had to rate the level of offense of the stimuli: they claimed that once a joke is funny it cannot be considered offensive, because “it’s clear that it’s a joke.”

fMRI results will be discussed in three sections: the outcomes related to DJ, those related to SI, and in the last section, the results of the ROI analysis.

### Disparagement Jokes

Overall, the main effect of DJ elicits activations in brain regions implicated in the three stages of humor processing: detection, comprehension, and appreciation. In particular, the activation in the bilateral SMG might be in relation to humor detection (Samson et al., 2008); the activation in the IFG, should be for humor comprehension (Goel and Dolan, 2001, 2007; Moran et al., 2004; Bartolo et al., 2006; Chan et al., 2012, 2013), whereas the activations in the bilateral cerebellum, the hippocampus, the insula, the ACC, and the amygdala bilaterally should be related to humor appreciation (Mobbs et al., 2003; Chan et al., 2016). Additionally, we also found activation in the caudate nucleus and thalamus bilaterally. The activation in the caudate nucleus has been found in gelotophobic participants exposed to hostile verbal jokes (Chan, 2016). Gelotophobia is a type of social phobia characterized by a fear of being laughed; gelotophobics are particularly sensitive to aggressive humor (Samson et al., 2011; Ruch et al., 2014; Chan et al., 2016). Chan et al. (2016) suggested that the activation of the caudate nucleus (a portion of dorsal striatum) might be in relation to its role in modulating “selective attention, planning, and effortful regulation of affective states.” In this view, we found activation of the caudate nucleus when participants perceive SI relative to N sentences, denoting the detection of a feeling of discomfort and the establishment of affective regulation (McGraw and Warren, 2010; Koszałkowska and Wróbel, 2019). Although we did not test gelotophobia and did not include non-hostile jokes, our behavioral data show that mainly DJ were perceived as less offensive than SI stimuli, suggesting that a sort of affective regulation process might take place when socially inappropriate funny stimuli are perceived.

An alternative interpretation derives from studies showing that the dorsal striatum is activated in participants that showed a desire to punish (de Quervain et al., 2004; Crockett et al., 2013). More specifically, de Quervain et al. (2004) conducted a PET study on punishment, in which participants could administer a punishment to unfair participants. Since punishment occurs to establish justice, the authors have interpreted the feeling that accompanied the punishment, as a sort of “sweet revenge,” as the administration of punishment is a “just revenge” toward defectors. As mentioned in the Introduction, disparagement jokes belittle individuals that are portrayed in a ridiculous manner. In some instances, the funny ending, although socially inappropriate, might generate a sense of justice toward the arrogance of the individuals targeted in the joke. Therefore, the activation of the caudate nucleus could be related to this feeling of sweet revenge coexisting with the feeling of mirth in disparagement humor.

This line of reasoning further suggests that disparagement humor might have much to do with the experience of schadenfreude. Schadenfreude is defined as an emotional reaction to misfortunes of others and it “is more likely when the protagonist is disliked, when actors pursue immoral goals and if they are responsible for their misfortunes” (Schindler et al., 2015, p. 1). Interestingly, the assessment of schadenfreude foresees a question like: “*how amusing/funny was the situation for you*?” In a recent study, Paulus et al. (2018) asked participants to rate the intensity of their amusement at the perception of a series of situations that elicit schadenfreude. They found activations in a network including, among others, the caudate nuclei bilaterally and the left thalamus (Paulus et al., 2018). Such a result might suggest the existence of a relation between disparagement humor and schadenfreude.

Relative to SI, DJ activated the bilateral ACC, the SMA, the precuneus, and the MFG. Relative to N stimuli, DJ activated the anterior insula, the putamen, and the entorhinal cortex. These regions have been previously found to be activated during humor appreciation (Goel and Dolan, 2007; Kohn et al., 2011; Shibata et al., 2014; Caruana et al., 2015, 2016; Liu et al., 2019; Wattendorf et al., 2019; Talami et al., 2020). Interestingly, some neuroimaging studies found activations in the ACC and the anterior insula during the threats to another person’s physical and social integrity (Beeney et al., 2011; Krach et al., 2011; Müller-Pinzler et al., 2016) and the left insula was also found activated in hostile jokes (Chan et al., 2016), suggesting that in DJ a feeling of mirth (and/or a desire to laugh) derived from the joke (e.g., SMA and precuneus) and the perception of the jokes’ social inappropriateness (e.g., ACC and insula) coexist.

Taken together, the anterior insula and the ACC are activated in DJ vs. baseline (both bilaterally), in DJ vs. N stimuli (the left anterior insula), in DJ vs. SI (the bilateral insula). These two areas are known to be related to empathy for pain (see Lamm et al., 2019 for a review). We can speculate that their activations reflected the feeling of empathy perceived in DJ.

### Socially Inappropriate but Not Funny Stimuli

SI stimuli activated a large network including frontal (inferior, superior, medial frontal gyrus, ACC), parietal (SMG and AG) temporal regions (STG) as well as the mesolimbic system (parahippocampal gyrus, amygdala, and the insula bilaterally). The activation of the cortical regions might be in relation to sentence comprehension and Theory of Mind for mental state attribution (Amodio and Frith, 2006; Olsson and Ochsner, 2008; Chan et al., 2016), whereas the mesolimbic system should be activated for affective and emotion regulation at the perception of the hurtful emotional content (Hamann et al., 2002; Benuzzi et al., 2004; Chan et al., 2016).

Relative to N stimuli, SI activated the right dorsal striatum (caudate nucleus) and the thalamus. In DJ, where the fun and the offense coexist, activations were found in the dorsal striatum bilaterally. We can speculate that the right caudate nucleus might be specific to negative emotions that arise at the perception of offensive sentences; whereas the bilateral activation in DJ could be related to a feeling of discomfort generated by the perception of something funny that is at the same time offensive.

Intriguingly, the contrast SI vs. DJ did not elicit any significant activation, as if the offense in SI and in DJ produces the same patterns of activations.

### Common Activations to Disparagement Humor and Socially Inappropriate but Not Funny (Regions of Interest Analysis)

This study also aimed at investigating the common brain network in disparagement jokes and socially inappropriate-not funny stimuli. Three functional ROIs were defined by using the contrast SI > N, namely the right thalamus, the left putamen and the left medial frontal gyrus. ROI analyses on the DJ > N contrast revealed a significant activation of these three regions, showing the existence of a common brain activity in DJ and SI with respect to neutral stimuli. In particular, brain activity in the medial prefrontal cortex has been associated with mentalizing processes (Amodio and Frith, 2006), third-person perspective-taking (Vogeley et al., 2004; Takahashi et al., 2009; Zahn et al., 2009; Müller-Pinzler et al., 2016), and empathy (Singer and Lamm, 2009; Bernhardt and Singer, 2012). Furthermore, we found activation of a portion of the left dorsal striatum (the putamen) and of the right thalamus, regions known to be relevant for the processing of socially inappropriate content (Schmahmann, 2003; Chan et al., 2016). Thus, the common regions between DJ and SI relate to a social cognitive complex process that goes from mentalizing to negative feelings arising in response to the perceived offense.

## Conclusion

In summary, this study provides evidence of the role of the dorsal striatum (in particular, the caudate nucleus), the thalamus, the ACC, and the insula in the processing of disparagement humor. In particular, the activation of the dorsal striatum denotes the presence of a feeling of discomfort in the perception of DJ and the establishment of affective regulation. This fMRI data are in line with the behavioral results that showed that participants considered DJ stimuli as less offensive than SI stimuli, suggesting that participants try to resolve a moral fight during the processing of DJ stimuli, as these are funny and at the same time offensive. An alternative explanation of the activation of the caudate nucleus is relative to a possible desire to “punish” the arrogance of the targeted characters that are portrayed in a ridiculous manner, something that has much to do with the experience of schadenfreude. Relative to SI, DJ activated the ACC and the anterior insula, regions related to empathy, suggesting that participants perceived the social inappropriateness of these jokes. Furthermore, while DJ activated the bilateral caudate nucleus, SI activated only the right part of this portion of the dorsal striatum. We speculated that the activation of the right caudate nucleus be specific to negative emotions (SI) whereas the bilateral activation be specific to the perception of the two conflicting feelings: the amusement for the funny ending and the discomfort for the offensive content.

Finally, ROI analyses allowed us to determine the three brain regions common in DJ and SI stimuli: the right thalamus, the left putamen and the medial frontal gyrus. Results suggest that the DJ and SI have in common a process of mentalizing and the perception of socially inappropriate content.

Overall, this study tried to investigate the nature of disparagement jokes by using an fMRI paradigm. Some limits and perspectives of the study deserve to be mentioned. First, the number of stimuli used in this study was small, as we included only 40 selected stimuli. In particular, the number of stimuli in the three categories was unequal and it could have had an impact on the results achieved. Another interesting question that could have not been explored in this study, concerns the gender difference in the perception of disparagement jokes. Sex differences in the perception of humor and in moral judgments have been already reported (Azim et al., 2005; Atari et al., 2020), therefore, this aspect deserves to be explored in future studies.

## Data Availability Statement

The raw data supporting the conclusions of this article will be made available by the authors, without undue reservation.

## Ethics Statement

The studies involving human participants were reviewed and approved by the Ethics Committee of the Province of Modena. The patients/participants provided their written informed consent to participate in this study.

## Author Contributions

AB, FB, PB and PFN devised the experiment. AB and FB prepared the stimuli. PB prepared fMRI paradigm. AB, FB, and LN tested participants. AB, FB, and DB ran the data analyses. All authors have contributed to the writing of the article.

## Conflict of Interest

The authors declare that the research was conducted in the absence of any commercial or financial relationships that could be construed as a potential conflict of interest.

## Publisher’s Note

All claims expressed in this article are solely those of the authors and do not necessarily represent those of their affiliated organizations, or those of the publisher, the editors and the reviewers. Any product that may be evaluated in this article, or claim that may be made by its manufacturer, is not guaranteed or endorsed by the publisher.

## Footnotes

1
www.pstnet.com

2
http://marsbar.sourceforge.net/

3
https://afni.nimh.nih.gov/pub/dist/doc/program_help/3dClustSim.html
